# Promising Therapies for Atrial Fibrillation and Ventricular Tachycardia

**DOI:** 10.3390/ijms232012612

**Published:** 2022-10-20

**Authors:** Andrei Alexandru Mircea, Mihaela Rusu, Elisa Anamaria Liehn, Octavian Bucur

**Affiliations:** 1 Victor Babes National Institute of Pathology, 050096 Bucharest, Romania; 2 Faculty of Medicine, Carol Davila University of Medicine and Pharmacy, 020021 Bucharest, Romania; 3 Department for Cardiology, Angiology and Internal Intensive Care, Medical Faculty, RWTH Aachen University, 5207 Aachen, Germany; 4 Institute for Molecular Medicine, University of Southern Denmark, DK-5230 Odense, Denmark; 5 Viron Molecular Medicine Institute, Boston, MA 02108, USA

**Keywords:** atrial fibrillation, ventricular tachycardia, vernakalant, vagal nerve stimulation, stereotactic ablative therapy, amiodarone, catheter ablation, electric cardioversion

## Abstract

Sudden cardiac death due to arrhythmias, such as atrial fibrillation or ventricular tachycardia, account for 15–20% of all deaths. Myocardial infarction increases the burden of atrial fibrillation and ventricular tachycardia by structural and electrical remodeling of the heart. The current management of new-onset atrial fibrillation includes electric cardioversion with very high conversion rates and pharmacologic cardioversion, with less a than 50% conversion rate. If atrial fibrillation cannot be converted, the focus becomes the control of the symptoms ensuring a constant rhythm and rate control, without considering other contributory factors such as autonomic imbalance. Recently, a huge success was obtained by developing ablation techniques or addressing the vagal nerve stimulation. On the other hand, ventricular tachycardia is more sensitive to drug therapies. However, in cases of non-responsiveness to drugs, the usual therapeutic choice is represented by stereotactic ablative therapy or catheter ablation. This review focuses on these newly developed strategies for treatment of arrhythmias in clinical practice, specifically on vernakalant and low-level tragus stimulation for atrial fibrillation and stereotactic ablative therapy for drug-refractory ventricular tachycardia. These therapies are important for the significant improvement of the management of atrial fibrillation and ventricular tachycardia, providing: (1) a safer profile than current therapies, (2) higher success rate than current solutions, (3) low cost of delivery.

## 1. Introduction

Sudden cardiac death due to arrhythmias accounts for 15–20% of all deaths [1]. Although efficient, the current medical and interventional therapies are far from the ideal solution. Both atrial fibrillation and ventricular tachycardias are increasingly affecting more people.

The Framingham Heart Study revealed that the prevalence of atrial fibrillation (AF) has increased three-fold over the last five years [2]. Moreover, the risk one may develop AF during his or her life has reached the proportion of 1 out of 4 people [3]. A review of the economic impact of the drug therapy in comparison with interventional therapy in AF has revealed a higher cost-effectiveness in the case of interventional therapies [4].

In the case of ventricular tachycardias (VTs), the perspective is somber. Current management of VTs is relying heavily on device therapy, such as implantable cardioverter-defibrillator (ICDs), catheter ablations, or old medications. However, it has been shown that VT recurrence is not improved by ICD implantation [5]. Furthermore, patients with high VT burden have a higher risk of mortality [5]. Lastly, an especially difficult problem is posed by the under-representation of women in VT therapy studies (RCTs) [6].

The RCTs tested whether catheter ablation, the last resort method for treating VTs, is an efficient therapy for VT.

The current options for the pharmacologic treatment of new-onset atrial fibrillation are flecainide, propafenone, or amiodarone, with less than 50% chance of success [7]. Atrial fibrillation ablation seems to be more efficient. However, it has a long-term success rate of only 50–60% [8]. Ventricular tachycardia became a concerning complication post-MI due to the tool restriction at our disposal. The ESC Guidelines’ drug recommendations included beta-blockers, sotalol, or amiodarone. When drugs fail, the only remaining options are ICD and catheter ablation. There are cases where even the invasive procedures fail or are rendered obsolete by the inaccessibility of the VT focus. The last resort in the management of drug-refractory ventricular tachycardia is catheter ablation [9].

Unfortunately, new therapies are needed to improve the outcomes and the side effect profiles. This review focuses on promising novel therapies that have at least two of the following three traits and can be used in cases where the currently accepted treatment fails:

1. Safer profile than current therapies.
2. As efficient or more efficient than current therapies.
3. Low cost of delivery.

Currently, there are only a few antiarrhythmics that have proven to be slightly more efficient than the existing antiarrhythmics. For example, vernakalant seems to be both more efficient and safer than amiodarone as a pharmacologic cardioversion agent [10]. Low-level tragus stimulation is a rhythm control method that has a low cost of delivery and a safe profile. Brinavess has already proven its efficacy in terminating atrial fibrillation. Adopted by the EMA and ESC guidelines in 2012, its approval is still pending for FDA. Furthermore, it has proven its higher efficacy to its counterpart (amiodarone) in medical cardioversion, as well as its safety profile [11].

On the interventional side, new non-invasive therapies have started emerging since 2020. An electrophysiologist team repurposed the vagal nerve stimulation method to reduce the length of atrial fibrillation episodes and to decrease atrial fibrillation burden [12]. Meanwhile, efforts were made to introduce a radiation oncology method (stereotactic ablative therapy) for refractory cases of ventricular tachycardia. The results surpassed the expectations previously set by the conventional catheter ablation therapy [13].

## 2. Atrial Fibrillation

**Figure 1:**
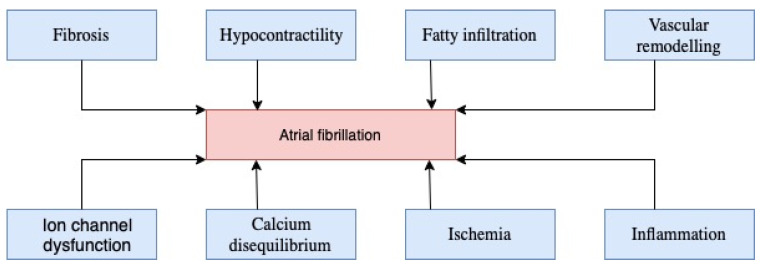
Contributing factors to atrial fibrillation.

Atrial fibrillation is a condition characterized by ectopic foci of depolarization at the atrial level. This supra-ventricular tachyarrhythmia transforms the atrial contraction into an inefficient one. Electrocardiographically, AF presents with (1) irregularly irregular rhythm (RR intervals), (2) absence of P waves, (3) irregular atrial activations. The mechanism underlying AF is multifold [14]. It includes fibrosis, hypocontractility, fatty infiltration, inflammation, vascular remodeling, ischemia, ion-channel dysfunction, and calcium disequilibrium (Figure 1) [14]. These factors contribute to both increased conduction and ectopic disruption and the hyper coagulable state of the AF [14].

### 2.1. Current Management of New-Onset Atrial Fibrillation

According to the ESC Guidelines [14], new-onset atrial fibrillation is managed with the ABC framework. Firstly, the patient is anticoagulated with either oral anticoagulants or vitamin K inhibitors. Then, better symptom control is ensured through rate and rhythm control. Lastly, comorbidities are controlled to prolong the patient’s life expectancy [14].

Rate control is pharmacologically achieved using mainly three drugs. There is no HR target, but rather an overall indication of less than 110 bpm [14]. The three drugs recommended for rate control by the ESC Guidelines [14] are beta-blockers, non-dihydropyridine (non-DHP) calcium channel blockers, and digoxin. The first line of rate-control therapy is a beta-blocker. In contrast, non-DHP calcium channel blockers not only improve AF symptoms as beta-blockers do but also preserve exercise capacity and reduce the BNP in patients with HFpEF [14]. Digoxin is employed only for severe, refractory cases.

Rhythm control is achieved with electric cardioversion in hemodynamically unstable patients or pharmacologic cardioversion. Electric cardioversion has a much higher success rate than pharmacologic cardioversion [9]. However, the side effects are potentially more dangerous.

The ESC’s pharmacologic management guidelines for new-onset atrial fibrillation in stable patients include: propafenone, amiodarone, flecainide, vernakalant (Table 1). However, for the first 24 h, it is preferred to adopt a wait-and-watch strategy, as 48% of new-onset atrial fibrillation will resolve spontaneously [14]. The current go-to options are either propafenone i.v., with a success rate of 59–78%, or flecainide, with a success rate of 43–89%. Amiodarone is preferred only for patients with heart failure. However, numerous studies [10,11,15,16,17] show that vernakalant is more effective than amiodarone or flecainide in all these cases.

Each of the pharmacologic rhythm control therapies acts through a different mechanism. Thus, amiodarone’s mechanism of action consists of blocking repolarizing potassium channels. It prolongs the duration of the action potential, as well as the effective refractory period. The uniqueness of this class III anti-arrhythmic drug is that it also blocks some calcium and sodium channels, and beta-adrenergic receptors. In comparison, flecainide and propafenone are type IC anti-arrhythmic drugs. They slow down the depolarization phase by blocking sodium channels. Their long-acting effect and narrow therapeutic index are the reason for the pro-arrhythmic effect. Lastly, vernakalant blocks atrial voltage-gated sodium channels in a dose and frequency-dependent manner and inhibits late sodium current. Moreover, it binds and inhibits the ultra-rapid potassium channels as well.

### 2.2. Novelty in the Pharmacologic Management—Vernakalant

Vernakalant (Brinavess) figures as a treatment of new-onset atrial fibrillation since 2012 in the ESC Guidelines. Having proven its efficacy, vernakalant still awaits FDA approval in the USA. The use in Europe demonstrates many characteristics of vernakalant, listed in Table 2. The studies conducted on vernakalant are listed in Table 3. 

Vernakalant (Table 3) is a 20 mg/g intravenous infusion recommended for new-onset atrial fibrillation (<7 days) or new-onset postoperative atrial fibrillation (<3 days). It is a pharmacologic cardioversion alternative to electric cardioversion. Vernakalant is administered in an initial 10 min infusion of 3 mg/kg. If the conversion does not occur yet, it will be followed by another 10 min infusion of 2 mg/kg spaced at 15 min [18]. 

Vernakalant preferentially acts on the atria by blocking currents of all phases, thus having a low proarrhythmic effect (vs. class IC). Firstly, it acts by blocking the ultra-rapid delayed rectifier and acetylcholine-dependent potassium channels which are located in the atria. Moreover, it blocks frequency and voltage-dependent sodium channels [18]. As frequency is high in atrial fibrillation, they are an ideal target. The low proarrhythmic effect is explained in part by inhibiting the late sodium current component. 

**Figure 2:**
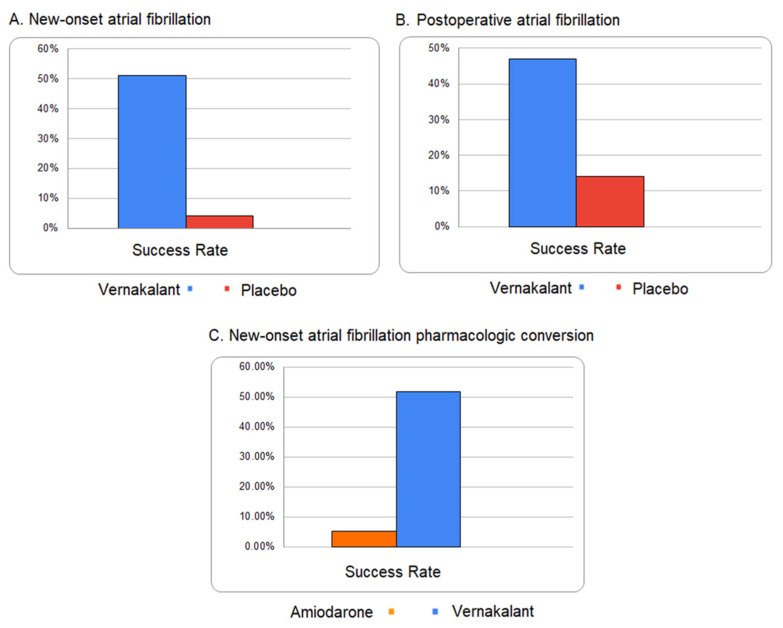
Comparative success rates for vernakalant (based on information provided by the European Medicines Agency [18]). (**A**) New-onset AFib—success rate of vernakalant versus placebo; (**B**) postoperative AFib—success rate of vernakalant versus placebo; (**C**) new-onset AFib—success rate of amiodarone versus vernakalant.

For treating new-onset atrial fibrillation, vernakalant proved its efficacy in three randomized, double-blind trials (ACT I, II, III) [5]. In the case of patients with atrial fibrillation < 7 days it led to conversion to sinus rhythm in 51% of the patients (vs. 4% with placebo). Also, it converted to sinus rhythm 47% of the postoperative cases (vs. 14% with placebo). Nevertheless, its efficiency was tested against an amiodarone infusion (gold standard for pharmacologic cardioversion). The results showed a 51.7% cardioversion in the case of vernakalant and only a 5.2% cardioversion with amiodarone [9] (Figure 2).

**Figure 3:**
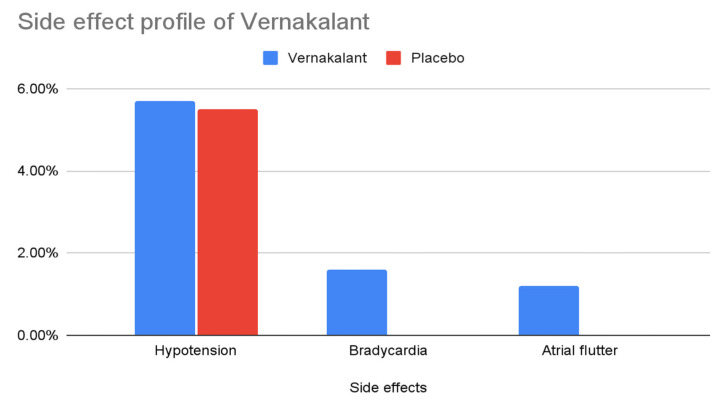
The side effect profile of vernakalant.

The side effects previously cited by the FDA as too risky are hypotension, bradycardia, and atrial flutter. Upon a thorough inspection, hypotension occurs in only 5.7% of patients treated with vernakalant and in 5.5% of patients receiving a placebo. These results change in a population of congestive heart failure patients (13.4% versus 4.7% with placebo). Bradycardia was shown to be rapidly responsive to atropine. There is 1.6% of occurrence in comparison with 0% for placebo. The atrial flutter occurs in only 1.2% of the cases treated with vernakalant [15] (Figure 3).

### 2.3. Novelty in the Symptom Control Therapy—Transcutaneous Electrical Vagus Nerve Stimulation for Atrial Fibrillation

Vagal nerve stimulation is a well-known, FDA-approved treatment for drug-resistant epilepsy and depression. After the 2018 FDA clearance for vagal nerve stimulation as an abortive therapy for migraines, an inquiry into whether this method might help with atrial fibrillation emerged [12]. 

Vagal nerve stimulation became a possible therapeutic tool as an alternative to the cervical ganglion block. It is well documented that atrial fibrillation has a component of sympathovagal imbalance [19]. Although the cervical block addressed this imbalance by suppressing the sympathetic output to the heart, the opposite method (increasing the vagal tone) was never researched in depth for atrial fibrillation [20].

In 2020, the TREAT-AF randomized control trial [12] demonstrated that low-level stimulation of the auricular branch of the vagal nerve at the tragus (LLTS) can have beneficial effects for atrial fibrillation patients. LLTS was shown to decrease the atrial fibrillation burden with chronic administration and to reduce inflammatory markers such as TNF-alpha. The active group received 1 h stimulation daily for 6 months. The results revealed a decrease in the total atrial fibrillation duration from 12 h in the beginning to 7 h after 6 months. If the initial atrial fibrillation burden amounted to 4.5%, by the 6th month it became only 2.0%.

By 2021, a Harvard-assembled team continued the efforts of the original Ohio University team, searching for biomarkers useful in LLTS therapy [21]. The P-wave alternans were used as a biomarker in this RCT. It was found that the patients whose PWA increased in response to the acute LLTS therapy experienced a greater decrease of AFib burden with chronic therapy. By contrast, patients without change in their P-wave alternans following acute LLTS therapy had a poorer response to chronic therapy.

Although LLTS cannot be used to terminate an atrial fibrillation episode, it has future potential in both improving the quality of life in patients with persistent atrial fibrillation and increasing their life-expectancy by reducing the atrial fibrillation burden.

### 2.4. Risks and Benefits—A Comparative Analysis

What happens when electric cardioversion fails to treat a newly-onset atrial fibrillation? Current guidelines indicate a pharmacotherapeutic conversion with flecainide, propafenone, or amiodarone. In a direct comparison on efficacy, vernakalant has higher efficacy than two of the available options, flecainide and amiodarone [10,11,15,16,17]. It takes less time to cardiovert with vernakalant than with the current therapies (~10 min). The side effect profile of amiodarone is vast, including hypotension, pulmonary alveolitis, tachycardia, bradycardia, heart block, and GI symptoms. In contrast, there are only three concerning side effects to vernakalant: hypotension, bradycardia, and atrial flutter (Table 4).

The current symptom control approach focuses on managing the heart (rhythm and rate) without concern for other contributing factors such as autonomic dysfunction. The single current therapy addressing dysautonomia is the cervical ganglion block for malignant tachycardia. There is no long-term therapeutic tool addressing the autonomic imbalance of atrial fibrillation for comparison. The vagal nerve stimulation method has an advantageous side effect profile (skin irritation, headaches, nasopharyngitis) [12,21]. Its long-term benefits in treating atrial fibrillation are considerable. They include (1) reduced cardiac remodeling, (2) reduced burden and duration of atrial fibrillation, (3) low cost, and (4) lowered levels of inflammatory cytokines.

## 3. Ventricular Tachycardia

One of the major causes of the sudden cardiac death (SCD) is ventricular tachycardia. VT is caused by an increase in ectopic activity in the ventricles [23]. There are two types of VT: (1) sustained monomorphic VT—which can degenerate into VF, and is frequently seen in a coronary ischemia setting, and (2) sustained polymorphic VT—which is triggered by either coronary ischemia or congenital/acquired QT prolongation. 

**Figure 4:**
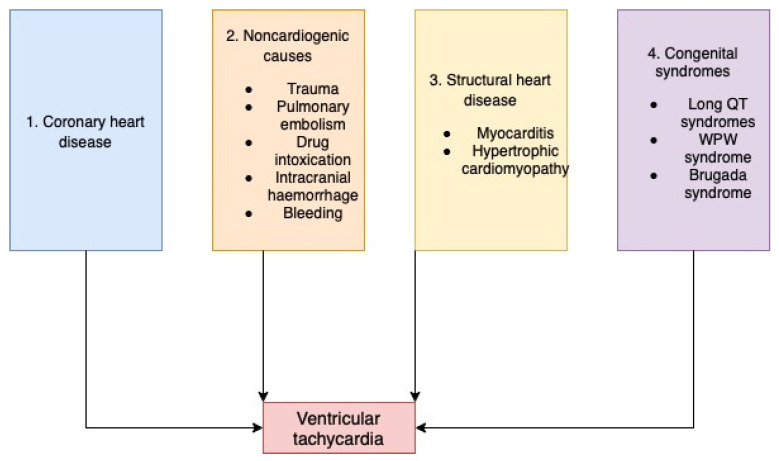
Etiology of ventricular tachycardia.

There are four major classes of pathophysiologic mechanisms underlying VT and SCD (Figure 4) [23]. First, the most common one is coronary heart disease. The second category of VT etiology from a frequency perspective is noncardiac. More precisely, VT develops secondary to trauma, pulmonary embolism, intracranial hemorrhage, drug intoxication, and bleeding. The third pathophysiologic mechanism is provided by an abnormal cardiac structure due to structural heart disease (hypertrophic cardiomyopathy [24], myocarditis). Lastly, congenital syndromes amount to only 5–10% of the total cases of VTs and SCDs. These include Brugada syndrome, Wolff–Parkinson–White syndrome, and long QT syndromes [23,25,26].

### 3.1. Current Management of Ventricular Tachycardia

According to the ESC Guidelines [9], ventricular tachycardia is managed in one of the three modalities: pharmacologic, interventional, or device therapy. The pharmacologic therapy for ventricular tachycardia revolves around beta-blockers, amiodarone, or sotalol. However, the latter presents with an unfavorable side effect profile on long-term administration. For the interventional side, the status quo is represented by catheter ablation. ICDs are the device therapy alternative.

The current pharmacologic treatment for ventricular arrhythmias is limited, without an impact on the survival rate and with potentially deleterious effects [9]. The first line drug is the beta-blocker, which acts by (1) preventing a sympathetic trigger of VT, and (2) inhibiting the excessive calcium release through the RyR channel. A concerning finding is that patients with more than two risk factors for shock were at higher risk of death when they were treated with beta-blockers [27]. The second option, namely amiodarone, has its use limited by its unfavorable side effect profile and patients discontinuing its use [28]. A previous trial highlighted that amiodarone was no better than placebo for survival benefits when LVEF < 35% [29]. The last option, sotalol, had a trial interrupted due to the pro-arrhythmogenic effects it bore on post-MI patients [30].

In contrast with pharmacologic therapeutics, ICDs are proven to improve survival rate and reduce mortality. A meta-analysis highlighted how ICDs reduce total mortality by 28% in patients with cardiac arrest or life-threatening ventricular tachycardia [31]. ICDs demonstrated their efficacy on a follow-up of 8 years [32]. However, there is a risk of 20% of inappropriate shock on a long-term scale [33]. Most importantly, the high up-front cost makes ICDs a limited therapy for several countries [14].

The current interventional method for VTs is catheter ablation. Its use is restricted to scar-induced VTs. Two studies showed that recurrency of VT in ischemic heart disease is successfully preventable with catheter ablation [34,35]. Nevertheless, this method is amenable for monomorphic VTs. While some polymorphic VTs can be managed with catheter ablation [36,37], not all the polymorphic, drug-refractory VTs are successfully treatable with ablation.

What complicates the situation of a stagnant development in pharmacologic therapies for VT are the drug-refractory cases. The current approach towards drug-refractory ventricular tachycardia is represented by a catheter ablation [9]. If this method fails, the last resort method is represented by surgical ablation. A current study shows a ventricular tachycardia burden decrease for typical ventricular tachycardia (not drug refractory ventricular tachycardia) of 99.6% [5].

### 3.2. Novelty in Pharmacologic Management—Azimilide, Ranolazine, Dofetilide (Table 5)

For a long time, VT remained one of the arrhythmias which had few pharmacologic treatment options. The one the guidelines indicated is amiodarone [9]. Recently, research has focused on enriching the pharmacologic options available for VT. Leaving the experimental drugs aside, some of the promising drugs to use as a potential treatment for drug-refractory VT are the three below. Of these, one has already proven its efficacy in the setting of drug-resistant VT.

**Table 5:** New pharmacologic therapies for VT.

Drug	Mechanism of Action	VT Benefits
Azimilide	Blocks rapid and slow delayed inward rectifier channels—class III	Reduces VT recurrence and hospitalizations in ICD patients
Ranolazine	Blocks Ca channels, delayed K^+^ channels, late sodium channels—class IB	Reduced ventricular arrhythmias Reduced VT episodes in ICD patients
Dofetilide	Blocks K^+^ channels—Class III	Reduced VT recurrence in post-MI patients Reduced electrical shocks and storms in ICD patients

The first drug is represented by azimilide. This drug acts by blocking both rapid and slow delayed inward rectifier channels [38]. Previous research showed that it significantly reduced the recurrence of VT, as well as hospitalizations in ICD patients [39]. Due to financial concerns the second SHIELD trial was interrupted, leaving this drug in the third stage of clinical trials [39]. Its side effect profile included only one percent of the treated patients who developed prolonged QT intervals [40,41].

Secondly, ranolazine proved efficacious for ventricular tachycardia. It is an antiarrhythmic capable of blocking numerous channels: calcium channels [42], delayed potassium channel [43], late sodium channel [44]. Ranolazine showed sustained reduction in ventricular arrhythmias during the MERLIN-TIMI 36 trial [45]. Moreover, patients with ICD benefited from ranolazine treatment by having their number of episodes reduced [46]. Both reduction of ventricular tachycardia and alleviation of ICD therapy were proven by a systematic review [44]. 

Ranolazine benefits from having its administration, indications, side effects, and contraindications already known [47]. The administration of ranolazine is made with a 375 mg/two dose per day, elevating itself to 500 mg/day × 2 after 2–4 weeks of administration. The maximum posology given is 750 mg/day. Ranolazine has been indicated in stable angina or unstable angina/NSTEMI. In the case of stable angina, its combination with amlodipine was shown to decrease the number of angina attacks as well as to reduce the number of nitroglycerin tablets taken [48]. The most concerning side effects include QT prolongation and drug–drug interactions. P-gp inhibitors as well as CYP3A4 inhibitors cause increases of serum ranolazine. For instance, careful titration is required for diltiazem administration with ranolazine, as diltiazem inhibits CYP3A4. Other drug interactions are represented by statins and digoxin, which have their plasma concentration enhanced by ranolazine. The same plasma concentration enhancement is seen with Metformin. On the other hand, the QT prolongation produced by ranolazine is dose-dependent (2.4 ms per 1000 ng/mL). The contraindications of ranolazine are class Ia or III antiarrhythmics (e.g., dofetilide, sotalol), CYP3A4 inhibitors (e.g., voriconazole, clarithromycin), renal failure (creatinine clearance < 30 mL/min), severe hepatic failure.

Lastly, dofetilide is already employed to treat VT in amiodarone-resistant patients [49]. One study showed similar results in regard to reducing VT recurrence post-MI between dofetilide and sotalol [50]. Furthermore, another paper demonstrated a reduction in ICD shocks and electrical storms when dofetilide was administered [51].

Dofetilide is currently used in the US, having its posology, indications, side effects, and contraindications labeled by the FDA [52]. Dofetilide doses are adjusted on a case-by-case basis, the doses being of 125, 250, or 500 mcg. Its indications are (1) the maintenance of normal sinus rhythm in patients that had a paroxysmal AF episode or an episode of atrial flutter, and (2) pharmacologic conversion of AF or atrial flutter. The serious side effects of dofetilide are sweating, vomiting, increased thirst, and QT prolongation. Therefore, patients with congenital long QT syndrome have a contraindication to this drug. Moreover, patients are hospitalized for three days to ensure correct titration of the drug. Dofetilide leads to torsades de pointes and VT. It is contraindicated in patients taking hydrochlorothiazide, verapamil, TMP-SMX, ketoconazole, as they increase the risk of torsades de pointes.

### 3.3. Novelty in Invasive Therapy for Drug Refractory VT—Stereotactic Ablative Therapy—SABR-HEART Trial

What had started in 2015 in Missouri as a last resort therapy for refractory ventricular tachycardia [53], has become a US clinical trial at MD Anderson Cancer Center for FDA approval [54]. Moreover, the method was adopted in Europe for selected cases of drug-refractory ventricular tachycardia. Once again, its efficacy was proven.

In 2015, Cuculich et al. demonstrated the ability to reduce the ventricular tachycardia burden with 99% for almost 4 years in five patients with ICD and drug-refractory ventricular tachycardia. Following this result, a study published in 2021 in Nature Communications [55] showed that the electrical conduction reprogramming caused by SABR is beneficial for the 19 patients suffering from ventricular tachycardia on heart failure. Thus, SABR increases the expression of conduction proteins in the heart, preventing reentry circuits.

Another consequence of this novel therapy is its global adoption. In the UK, seven patients with ventricular tachycardia underwent the same SABR protocol with a decrease in the ventricular tachycardia burden by 85% at a 6-month follow-up [56]. Another trial was conducted on six patients with refractory ventricular tachycardia at Verona University [57]. Five patients experienced one or no ICD shocks in the following 6 months.

### 3.4. Risks and Benefits for Treating Drug-Refractory VT—A Comparative Analysis

The treatment of drug-refractory ventricular tachycardia can shift to a safer and better procedure (Table 6). The most frequent complication of the current management using catheter ablation is vascular complications. Patients develop femoral pseudoaneurysms, groin hematomas, arteriovenous fistulas. On the other hand, stereotactic ablation therapy has no vascular complications. Its benefits continue further. There is no need for anesthesia. Thus, ventricular tachycardia at extreme ages is facilitated. The catheter ablation is limited by the depth of the aberrant circuits. However, the stereotactic method allows to burn the aberrant circuits deep within the interventricular septum.

### 3.5. Genetics of Ventricular Tachycardia versus Genetics of Atrial Fibrillation

Although these conditions frequently have an underlying anatomic or functional substrate, recent studies showed that they both can have a genetic component as well. For instance, familial AF has mutations in genes such as (1) KCNQ1—coding for an ion channel, (2) NPPA—coding for a cardiac peptide, (3) TBX5—coding for a transcription factor, (4) MYL4—a motor protein, or (5) TTN—coding for titin [58]. On the other hand, monogenic factors contributing to congenital syndromes with VT include mutations in genes encoding (1) the sodium channel protein (SCN5A), (2) the potassium channels (KCNQ1 and KCNH2), (3) calcium channel (RYR2) [59]. Thus, the calcium channel mutation is the defining trait for catecholaminergic polymorphic ventricular tachycardia, while the potassium channel mutations are present in long QT syndromes. The sodium channel mutation is seen with Brugada syndrome [60]. Familial AF and VT amount the least from the current population suffering from such diseases. However, genetic therapy is a promising avenue for these congenital etiologies.

## 4. Conclusions

All in all, these new medical and non-invasive therapies promise that the future of electrophysiology is no longer reliant on invasive procedures or old drugs, such as amiodarone. The cost-efficiency is higher with the discussed new promising methods. For instance, LLTS requires minimal costs of delivery. Proving its efficacy in cardioversion, vernakalant becomes a competitor to the conventional electric cardioversion. On the pharmacologic front, VT has started to benefit from new therapies that are currently employed as a second line solution. Dofetilide and ranolazine are potent competitors for a first line drug. In contrast, azimilide may open new therapeutic opportunities once the trials are re-initiated. Lastly, the clinical trials of stereotactic ablative therapy for arrhythmias demonstrate not only an interest for developing such methods, but also an acknowledgement of its performance by both government and medical field representatives.

All these new promising therapies and others will have a huge impact on clinical practice paradigm and society, preventing an ever-aging population from reaching high burdens of AF and VT. It is expected that what was once considered an epidemic [61] will have little-to-no impact on public health in the future.

## Figures and Tables

**Table 1:** Mechanism of action for rhythm control drugs used for new-onset AF.

Drug Name	Mechanism of Action
Amiodarone	Blocks repolarizing K channels
Flecainide and propafenone	Blocks depolarizing Na channels
Vernakalant	Blocks atrial voltage-gated Na channels and ultrarapid repolarizing K channels

**Table 2:** Summary of vernakalant properties (based on information provided by the European Medicines Agency) [18].

Drug Name	Vernakalant
Administration	3 mg/kg i.v. for 10 min After 15 min, 2 mg/kg i.v. for 10 min
Indications	New onset atrial fibrillation (<7 days) Postoperative atrial fibrillation (<3 days)
Efficacy (vs. placebo)	51% (vs. 4%) for new-onset atrial fibrillation 47% (vs. 14%) for postoperative atrial fibrillation
Side effects	Hypotension (5.7% of cases) Bradycardia (1.6% of cases) Atrial flutter (1.2% of cases)
Contraindications	Congestive heart failure Use of antiarrhythmics (class I and/or III) within the 4 h window before and after vernakalant administration Valvular heart disease Acute coronary syndrome in the last 30 days QTc > 440 ms

**Table 3:** Current and previous studies of vernakalant (according to the clinicaltrials.gov website).

Study	Primary Outcome Studied	Location
Vernakalant versus Ibutilide in Recent-Onset Atrial Fibrillation	Time in minutes until conversion to sinus rhythm (measured from the start of the first study drug administration)	Medical University of Vienna, Department of Emergency Medicine
A Study of the Efficacy and Safety of Vernakalant Hydrochloride (MK-6621) in Patients with Atrial Fibrillation	Proportion of patients with treatment-induced conversion of atrial fibrillation to sinus rhythm	Advanz Pharma
RAFF4 Trial: Vernakalant vs. Procainamide for Acute Atrial Fibrillation in the Emergency Department	Conversion to sinus rhythm for a minimum duration of 30 min	Hopital Du Sacre-Coeur, Montreal; Montreal Heart Institute, Montreal; Sunnybrook Hospital, Toronto
Predictive Factors to Effectively Terminate Paroxysmal Atrial Fibrillation by Blocking Atrial Selective Ionic Currents	Electrocardiographic-based spectral parameters of atrial fibrillatory activity (dominant frequency) associated with successful or unsuccessful cardioversion in both groups of patients.	Hospital Clinico Universitario San Carlos, Madrid
Study of Normal Conditions of Use, Dosing, and Safety of Intravenous (IV) Administration of Vernakalant	Number of participants experiencing significant hypotensionSignificant hypotension is defined as: symptomatic hypotension with systolic blood pressure (BP) < 90 mmHg, requiring treatment with vasopressors Number of participants experiencing significant ventricular arrhythmia Number of participants experiencing significant atrial flutter Number of participants experiencing significant bradycardia	University Hospital of Vienna, Austria
A Study Comparing Vernakalant Therapy to Amiodarone Therapy in Acute Management of Recent Onset Atrial Fibrillation	Number of participants discharged from the emergency room (ER) to home, home-equivalent, or long-term care facility (LTCF) within 12 h from randomization	Multicenter
A Phase III Superiority Study of Vernakalant vs. Amiodarone in Subjects with Recent Onset Atrial Fibrillation	Proportion of subjects with conversion of atrial fibrillation to sinus rhythm within 90 min after the start of infusion	Royal Adelaide Hospital, Australia
Vernakalant (Oral) Prevention of Atrial Fibrillation Recurrence Post-Conversion Study	Time to first documented recurrence of symptomatic sustained AF Safety assessments—vital signs, safety laboratory assays, ECG parameters, physical examinations, and frequency of adverse events	Royal Adelaide Hospital, Australia

**Table 4:** Risks and benefits for new-onset atrial fibrillation and symptom control therapeutics.

Therapy	New-Onset Atrial Fibrillation Therapies			Symptom Control Alternative Therapies
	Electric Cardioversion [14]	Amiodarone Cardioversion [22]	Vernakalant Cardioversion [18]	LLTS [12,21]
Risks	Sedation-related complications, ventricular fibrillation, bradycardias, tachycardias (atrial flutter), torsade de pointes	- Hypotension, pulmonary alveolitis, tachycardia, bradycardia, heart block, GI symptoms	- Hypotension - Bradycardia - Atrial flutter	- Skin irritation - Headaches - Nasopharyngitis [12,21]
Benefits	- High success rate - Useful in hemodynamically unstable patients - Rapid conversion without requiring any imaging (<48 h)	- Useful in patients with heart failure	- Higher efficacy than amiodarone or flecainide (currently used) - Useful in postoperative AFib and new-onset atrial fibrillation (3–7 days) - Rapid termination of atrial fibrillation (conversion in less than 10 min from infusion)	- Reduces cardiac remodeling - Reduces burden and duration of AFib - Low cost - Lowers inflammatory cytokines
Success rate	90%	44% (within several days)	50% in less than 10 min from administration	Decreased atrial fibrillation burden (4.5% to 2%) and atrial fibrillation duration (12 h to 7 h)

**Table 6:** Risks and benefits for drug refractory ventricular tachycardia therapeutics.

Therapy	Drug Refractory Ventricular Tachycardia	
	Catheter Ablation [9]	Stereotactic Ablation Radiotherapy [49,50,51,52,53]
Risks	Vascular access complications (femoral pseudoaneurysm, groin hematoma, arteriovenous fistula) Transient ischemic attack/stroke Conduction system damage (left bundle branch block, atrio-ventricular block) No back-up method if it fails (the alternative surgical ablation has too high a risk)	No vascular access complications
Benefits	High success rate Previously established method	Requires no anesthesia Allows for dose modulation for hard to reach or sensitive areas Back-up method if catheter ablation fails Only one application required, reducing the dose or stopping antiarrhythmics
Success rate	Ventricular tachycardia burden decrease of 99.6%	Ventricular tachycardia burden reduction between 86% and 99%

## Data Availability

Not applicable.
